# Work-family balance by women GP specialist trainees in Slovenia: a qualitative study

**DOI:** 10.1186/s12909-016-0551-2

**Published:** 2016-01-28

**Authors:** Davorina Petek, Tadeja Gajsek, Marija Petek Ster

**Affiliations:** Department of Family Medicine, Faculty of Medicine, University of Ljubljana, Poljanski nasip 58, 1000 Ljubljana, Slovenia; Community Medical Center Maribor, Ulica talcev 9, 2000 Maribor, Slovenia

**Keywords:** Workload, Family, Burnout, Empowerment, Coordination

## Abstract

**Background:**

Women physicians face many challenges while balancing their many roles: doctor, specialist trainee, mother and partner. The most opportune biological time for a woman to start a family coincides with a great deal of demands and requirements at work. In this study we explored the options and capabilities of women GP specialist trainees in coordinating their family and career.

**Methods:**

This is a phenomenological qualitative research. Ten GP specialist trainees from urban and rural areas were chosen by the purposive sampling technique, and semi-structured in-depth interviews were conducted, recorded, transcribed and analysed by using thematic analysis process. Open coding and the book of codes were formed. Finally, we performed the process of code reduction by identifying the themes, which were compared, interpreted and organised in the highest analytical units – categories.

**Results:**

One hundred fifty-five codes were identified in the analysis, which were grouped together into eleven themes. The identified themes are: types, causes and consequences of burdens, work as pleasure and positive attitude toward self, priorities, planning and help, and understanding of superiors, disburdening and changing in specialisation. The themes were grouped into four large categories: burdens, empowerment, coordination and needs for improvement.

**Conclusion:**

Women specialist trainees encounter intense burdens at work and home due to numerous demands and requirements during their specialisation training. In addition, there is also the issue of the work–family conflict. There are many consequences regarding burden and strain; however, burnout stands out the most. In contrast, reconciliation of work and family life and needs can be successful. The key element is empowerment of women doctors. The foremost necessary systemic solution is the reinforcement of general practitioners in primary health care and their understanding of the specialisation training scheme with more flexible possibilities for time adaptations of specialist training.

## Background

In Slovenia, the feminisation of the medical profession has been present since the middle of the 1960s. Recent data shows that 65 % of doctors in Slovenia are women, while the ratio of male and female doctors in general practice (GP) is 1:5 [1]. Currently, women GP specialist trainees in Slovenia represent 73.5 % of all GP specialist trainees (278 women out of 378 all GP specialist trainees). A similar ratio of women doctors can be found in other developed countries [2–4]. 113 new GP trainees started specialist training in the year 2014, and 83 started in 2015.

In Slovenia, four-year specialist training and the final specialist exam are compulsory for licensed independent work as a family physician with his/her own list of patients. The training consists of two parts of rotations: two years in clinical specialties (mainly in hospitals) and two years in family medicine practices. Throughout this time, the trainees are regularly included in performing emergency services. Each trainee is supervised by an advisor (head of training) during the entire training and by the immediate supervisor appointed for each rotation [5]. Often, due to the lack of GPs, they also work as substitute GPs in distant locations where the immediate supervisor is not available for direct consultation.

According to the literature, the majority of women doctors do not choose between a career or family, but aim to have both. They often contemplate on how to coordinate their family and career when choosing their specialisation [3, 4, 6]. Some of them work part-time; many choose a specialisation that allows them to better coordinate both, while others change their specialisation mid-course in the interest of their family or children [3, 7]. Consequently, women doctors dominate in primary health care, where coordinating family and career is allegedly easier and the work is less physically demanding.

The conflict between the demands of their work and family needs, which is considered one of the biggest sources of stress for employees, is also noted by men doctors, but to a much lesser extent than women doctors [4, 8]. Robinson states in her review paper that 26.4 % of women doctors recognised a strong conflict of roles, whereas only 6.1 % of men doctors recognised the same conflict [7]. It is historically accepted in society that men work full-time and that the needs of their job come before the needs of their family.

Women doctors therefore report about stress more often [7]. Long-term stress can lead to burnout, depression, mental stress, insomnia, physical problems and disease. Depression is more common amongst women than men doctors, but approximately the same as is true of the general population [7, 9, 10].

Conflicting information is given in the literature as to the proportion of burnout among women doctors. An important fact to consider is that only a few studies have been conducted that analyse burnout in terms of gender. Mc Murray and co-workers showed in their study that women doctors have a 60 % greater likelihood of reporting burnout in comparison with men doctors [11]. A similar study including 1426 women and men doctors was conducted in Netherlands, which revealed no significant differences in the proportion of burnout in both genders [12].

A number of studies clearly show that burnout is the highest among specialist trainees, due to their long working hours, the vast amount of clinical knowledge they need to acquire, their inexperience, and difficulties in coordinating their family and career [10, 13, 14].

Between 2008 and 2009, a cross-sectional study was also conducted in Slovenia researching burnout in GP specialist trainees [15] that showed a high prevalence of burnout among women trainees, comparable to the prevalence of burnout amongst doctors-specialist trainees in other studies [10, 13, 14]. Due to a lack of GPs in Slovenia, the workload per GP is very high. Together with Slovakia, Slovenia has, the lowest number of GPs per 1000 inhabitants in Europe (0,48 GPs per 1000 inhabitants in Slovenia, in comparison to 1,7 GPs in Belgium) [16]. Trainees are also subjected to high burdens during their rotation in the practices [17]. Quality improvement of the training programme and requirements for regular trainee assessment bring new assignments for trainees. Nevertheless, very few women trainees apply for the prolongation of the training programme.

The aim of this study was to explore the options and capabilities of women GP specialist trainees in their coordination of family and career.

Specific objectives of the study were to determine the perceived burdens of women GP specialist trainees, the consequences arising from these burdens and the practical solutions or life adaptations they have implemented to coordinate their different roles.

The study was approved by the Republic of Slovenia National Medical Ethics Committee on 8 April 2014 under the number 48/04/14.

## Methods

A phenomenological theoretical framework was used enabling the determination of experience and its meaning in a specific time and place. We looked at the personal experience - the meaning and decisions of the participating trainees when coordinating their burdens. This method was chosen for its inductive nature, providing valuable insight into various perspectives of the study participants. The data was gathered via semi-structured in-depth interviews.

The following research question was asked: » How do women GP specialist trainees coordinate their family and career? «

### a) Sample

The sampling technique was purposive: ten participants were chosen from the list of women GP specialist trainees, obtainable from the national coordinator of the training programme. Only trainees with at least one child were eligible for inclusion. We included trainees in different years of their specialist training, and from different urban or rural home and work locations. We also included trainees working within a bigger team where the immediate supervisor is at close access and trainees working in distant, solo practices.

We conducted 10 in-depth interviews with women GP specialist trainees between the ages of 28 – 36 who live with a partner and have 1 to 4 children. They work in general practices in different parts of Slovenia. Their demographic characteristics are described in Table 1. All of the participants gave their informed consent for participating in this study.

**Table 1:** Demographic characteristics of participants

Trainee	Age (years)	No of children	Year of training^a^	Location of practice	Distance from work (km)	Other GPs at the same location
1	36	3	3.5	urban	26	yes
2	38	3	2.5	urban	4	yes
3	32	2	3	rural	3	no
4	37	1	3.5	urban	20	no
5	36	4	3.5	rural	20	no
6	35	2	2	rural	1	no
7	30	2	1	urban	1	yes
8	31	1	1.5	uban	1	yes
9	35	2	3.5	rural	37	no
10	33	3	3,5	Urban and rural	5,5 KM	Yes/no^b^

Legend

^a^number of years of rotation from the start of the training programme

^b^works on two locations

We interviewed and included them in the study until the saturation of gathered data was reached or until no new codes were derived. Saturation was met with interview number 10.

### b) Data gathering

We asked eight basic questions which we supplemented with sub-questions where needed (in brackets). The interviews were all conducted by one person (TG - specialist trainee herself) applying a consistent interview technique. Five interviews were performed at the health-care centres where the participants worked, three were performed at the Department of Family Medicine in Ljubljana and two were performed at informal locations.

The following open-type questions were used:

- Please tell us about your burdens as a GP specialist trainee (work in the practice, on-call duty, exams, modular course assignments, children, housework).
- How satisfied are you with spending time with your family in terms of quality and amount?
- How do everyday strains impact your ability of reflection, concentration and your work motivation?
- How do everyday strains impact your behaviour or communication with your family and your patients (kindness, patience, irritability, negligence…)?
- How do you coordinate your family and career (pregnancy planning, number of children, working part-time, the choice and time of specialisation training)? Who assists you and how (partner, parents, colleagues, superiors)?
- How do you look after yourself and your needs?
- Which changes in the health care system do you consider necessary for improving the position of women GP specialists in training? What changes in your personal or family life would disburden you?

The interviews lasted from 30 to 60 min. All interviews were audio-taped and transcribed verbatim. The accuracy of the transcriptions was checked and compared with the audiotape. In respect of the participants’ privacy, their names or identities are not disclosed in this text.

### c) Data analysis

After repeatedly reading the transcripts and becoming familiar with the text, two researchers (TG and DP) independently coded the text by inductive thematic analysis codes deriving from the text [18]. In the cases of differences in the codes, the agreement was achieved through discussion of all three researchers. The first part of the process was finished by creating the initial coding book. The next stage was the reduction of codes and their sorting into clusters (themes) by similarities in meaning, and further on into categories based on the relation and linkage of the lower hierarchical level.

The text citations and created codes were entered in an Excel table, where the codes were then combined together into themes and categories.

## Results

One hundred fifty-five codes were identified in the first analysis, which were then reduced and gathered by similarities into codes of higher rank and then grouped into 11 themes and into 4 categories. The identified themes were the following: types of burdens, causes of burden, consequences of burdens, work as pleasure, positive attitude to self, priorities, planning, help, superiors, disburdening, changes in specialisation The themes were gathered into four categories: burdens, empowerment, coordination, needs. Table 2 shows the results of the interview analysis.

**Table 2:** Results of qualitative analysis with categories, themes and number of codes

Categories	Themes	Subthemes	No of codes
Burdens	Types	Burdens at work	14
		Burdens in specialisation	8
		Burdens at home	4
	Causes	Insufficient qualification	4
		Junior work status	8
		Collective self perception	10
	Consequences	Dissatisfaction, feeling guilty	4
		Neglecting self- needs	7
		Exhaustion/burnout	17
Empowerment	Work as pleasure		3
	Positive attitude toward self	Optimism	3
		Feeling successful	5
		Self-care	25
Coordination	Priorities		4
	Planning a family and family life		6
	Help	Partner	6
		Parents	3
		Colleagues and the team	7
Needs for better coordination	The understanding of superiors		4
	Disburdening		8
	Changing in the specialisation/training organisation		5

### A Burdens

#### Types of burdens

- Burdens at work

The most important perceived burdens at work were the number of patients and the fast pace of examining them, cover shifts, when they have to cover for any absent colleagues and simultaneous emergency care, parallel to regular practice work.

*»…but the number of patients I have to see and knowing that there isn’t enough time to discuss anything with them, except their main problem. Ok, you have a burning sensation in your eyes, ok, here are some eye drops, come back for a check up, come on this date, take care, goodbye, next please. It is almost like a mass production of patients. » (P10)*

*» Chaos always prevails when you are on sick leave, I also find it hard to be at work all day long and cover for the entire clinic. If I were to call in sick, other mothers would have to come in and cover for me. « (P4)*

*»Working in the clinic is very burdensome, because I have been working independently in my clinic since day 2 and my patients all have appointments. In parallel, I also work in emergency care. Every transport to and from the hospital takes about an hour, hour and a half. When I get back, I have no time for lunch, because there is a bunch of patients waiting, so it’s all very stressful. « (P6)*

b) Burdens in specialisation training

The specialisation training requirements represent a critical burden. There is often insufficient time to meet all of the specialisation requirements, such as modular course assignments and study time.

c) Burdens at home

At home, housework, and the needs and expectations of their children await them.

*» You come home and your two children are waiting for you, as well as all of the housework, your specialisation work, which is usually left for late at night when the children fall asleep and your husband settles down in front of the TV, so yeah, there is a lot to do. « (P9)*

#### Causes of burdens

- Insufficient qualification

In their work, the residents mentioned burdens due to insufficient experience, uncertainty and lack of clinical knowledge, usually applied to specific tasks which are rarely performed, such as work in the anticoagulant clinic, work in the paediatric clinic, emergency care and the nursing homes. They are often left to themselves without the possibility of consulting their supervisor.

*» I find working at the clinic very demanding, when I have to work alone, when my mentor is not in the next room and I cannot consult her in case of a problem, that is, when I am covering in other clinics, then I do the best that I can or I call other doctors who I know are on duty at that time, which is all very disorganised, I should always be able to have someone to call, someone to rely on.« (P3)*

b) Junior work status

They are set apart from their colleagues by the generation gap, because older colleagues are not familiar with the specialisation training scheme and the status of young trainees, therefore they do not understand their troubles.

*»…there is also this sense of being the youngster, the last one to come work here, it’s not your place to impose your rights, we all went through it, you know. « (P9)*

### There are untold expectations from senior colleagues

*» I have a feeling that it’s expected of us, even though nobody says it outright, that you will give your all during your specialisation training, that you will excel, that you will do your best in everything, to form a good image, a reputation for collaboration in the future« (P9)*

c) Collective self-perception

They also pointed out that they treat patients in too much detail and too slowly. They perceive themselves as too self-sacrificing, too helpful, too obliging, exposed to the high expectations of others.

*» I think that in general practice we have the type of character of being helpful, wanting to help others and wanting to act in this way, so we often, if I speak for myself as well, we often go beyond our needs and the needs of our family, unfortunately, but it’s not right.« (P9)*

### Self-expectation makes delegation of work more difficult

*» I think that as general practitioners, we all feel that we have to do everything ourselves, that we can do everything, that we are capable of doing everything by ourselves and therefore, I must do everything myself. I think that we often forget that it is not a crime to share the work. « (P10)*

#### Consequences of burdens

- Dissatisfaction, feeling guilty

The different burdens leave specialist trainees with not enough time for their family and children. As a consequence, they have feelings of guilt regarding their family and children.

*» It often happens that my partner takes care of the kids more often that I do and that’s why I feel guilty, but I really don’t know…in the evening, when I come home, I try to compensate, look, I was on call duty, I wasn’t here, I have to spend time with them. « (P6)*

Feelings of guilt also spring up in the relationship towards the organisation of work, especially in the case of absence due to sick leave. They usually avoid taking any sick leave.

*»When my daughter was very ill in Year 1, I had a constant dilemma, I constantly felt guilty because of my work and the organisation of work, because I was absent a lot, because I had no help. « (P1)*

b) Neglecting self needs

They are unsuccessful in reserving time for themselves. They neglect their own needs, even the most basic ones, such as sufficient sleep, food and physical activity.

*» I know that I advise many of my patients the opposite of what I do myself, that’s a fact. A fact. « (P9)*

c) Exhaustion (burnout)

The specialist trainees named the following symptoms of burnout syndrome: feeling of exhaustion, chronic fatigue, insomnia, lack of motivation, poor concentration, mistakes at work, mood swings, irritability, the inability to quickly calm down or relax, low stress tolerance, apathy, susceptibility to illnesses, and psychosomatic symptoms: headache, abdominal pain.

*»…these symptoms occur anyway: headache, abdominal pain, fatigue, mood swings, fatigue from lack of sleep, I am extremely tired but cannot fall asleep. « (P9)*

Nevertheless, at work, they maintain a professional attitude: they have excellent self-control, they are polite etc. But at home they are often irritable, ill-tempered and impatient, especially with their children, but also towards their partner.

*» I am often cranky, snappy and tired when I get home. You come home where everything comes off the shoulders and you can be who you are and not maintain a pose like at work, where you have to be professional and polite and patient, so you lash out at people at home who don’t deserve it, your husband, children and the dog. They don’t deserve it. « (P3)*

#### B Empowerment

Although they are faced with high levels of stress at home and at work, they find the power within themselves to take control over their lives and actively cope with burdens and stress.

#### Work as pleasure

They feel satisfied and very motivated in their work. Their work, or satisfaction in their work, also serves them as a source of energy for other areas of life.

*» Everything I do at work or anywhere else with a sense of satisfaction and enjoyment doesn’t only tire me out, but also rewards me with energy, and so the strength to go on. This is how I am partially relieved of my burdens. « (P1)*

#### Positive attitude towards self

An important part of empowerment, which some trainees are skilled at, is a positive attitude towards themselves, and knowing their own limits and needs.

Specialist trainees are optimistic and believe in the transiency of the current state of affairs.

*» I see that I can hang in there by having a definite goal, a set timeframe till the exam. I hope that as a resident I will know how to manage my work conditions and other things so that working in my own clinic will be different. « (P9)*

A successful resolution of problems and success in the work place both contribute to the feeling of self-confidence and competency.

*» I always set things out in a broad sense. I am also a lecturer at the Red Cross and I lead trainings, which is an additional burden. I often ask myself if it’s necessary. Do I enjoy it? Also, I found the preventive project interesting, so I agreed to be the regional doctor in charge of the preventive project; they have appointed us for the colorectal cancer screening programme in our region, this is good for the soul, it’s also a burden, but it energises you. « (P10)*

They take care of their basic needs of sleep, healthy eating and exercise. They find a source of relaxation in spending time with their friends, partner, family, and in physical activities, which they try to do at least twice a week.

*» I try to meet up with my friend who lives in the village every Thursday, we go for a coffee or a fruit cup and we have that hour to ourselves.. Then I come home and dive in with full power. I always have it in the back of my head that I need to do something for myself as well, even though I need to speed things up afterwards to finish everything I had set out. « (P10)*

They find a source of happiness and pride in hobbie*s.* They also practice meditation, attend self-help groups in the evenings, when they are tired and need time for themselves to relax in silence, with music or meditation.

*» I am often the first one to give out the sign, I lie down and do nothing, because I need to unplug, I often need only half an hour or a couple of minutes to calm down, unplug, regenerate, and then I get up and do whatever else needs to be done.”(P1)*

#### C Coordination

### Priorities

The trainees stressed the importance of good organisation, both at work and at home, and sensible life choices.

### Planning a family and family life

They try to spend time with their children and actively participate in activities with them.

*» I do things that make me happy and I try to involve my whole family and children. I like gardening, we have two gardens at home and we all go outside, even though my children are small, and we do it together.« (P9)*

They plan their family very carefully. Becoming pregnant is often a decision influenced by the time of their specialisation training.

*“If I had a different job and more time for myself, more time for my family, I would definitely opt for another child, but at the moment I am only strongly considering whether to have another one.” (P4)*

### Help

Having an understanding and supportive partner for moral support and assistance is very important. The partner somewhat relieves them of burdens at home.

*» We do everything ourselves. We divide the chores. « (P4)*

Their parents help out a great deal in looking after the children in the afternoons, helping with housework. They also often take care of the children when they are ill. The trainees who have no or minimal support from their parents stated a greatly reduced possibilities in coordination. They try to coordinate with a babysitter.

*» My partner has less time than me, he cannot help out much, but the grandparents can, because they are both retired for two or three years now. We live in the same house, grandpa takes them to the nursery, grandma takes care of the rest, I have a lot of support and I cannot image what I would do without it. « (P5)*

At work, the solidarity of their colleagues and the understanding of their superiors is a big help to the trainees. An important role in the help and moral support is provided by the nurse at the clinic.

*» I am lucky that I have such a great nurse, supportive, always helping me out. She notices if I’m stressed out and she takes care of things.” (P10)*

#### D Needs for better coordination

The majority of them think that changes should be implemented on the health care system level that could disburden women specialist trainees with children. They think that motherhood should factor in the work consideration.

#### The understanding of superiors

Superiors should be more understanding towards women doctors with small children, in particular in the case of absence from work.

*» Things should be more family-oriented, in terms of not being frowned upon, without having the feeling that taking advantage of part-time work is not something that is frowned upon in your own work place. «(P9)*

#### Disburdening

An increased number of doctors in the health care system would relieve burdens of trainees, enabling them to focus more on their work and education. It would be easier to get covers for shifts and go on sick leave when their children fall ill.

*» Taking sick leave is very important, I want to be with my child when he’s ill, the clinic should provide a cover and I should be able to take sick leave so that my child could go to preschool normally, because he doesn’t at the moment - he would be ill too often.« (P4)*

Their suggestions include: being exempt from on-call duty, night and day, weekend on-call duty and afternoon shifts; the possibility to extend the specialist training from 6 to 12 months for mothers with children without having to submit an application; part-time work if it does not have a negative financial impact.

#### Changes in the specialisation-training organisation

They suggest that more time should be consecrated to rotations at the pre-hospital emergency care or emergency care, which would enhance their skills for independent on-call duty or emergency care work.

**Fig. 1:**
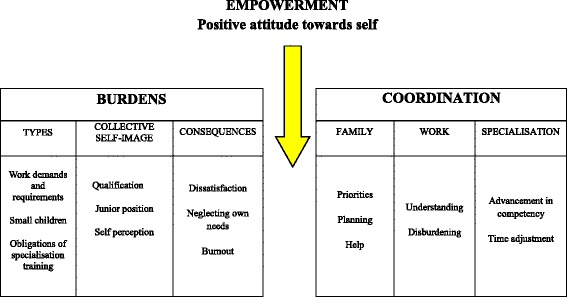
Coordinating burdens by empowering women doctors as an intermediary factor

They would like to have the possibility of additional training according to their own needs and priorities, and the possibility of permanent consultation with experienced co-colleagues (Fig. 1).

## Discussion

The study results clearly show that the specialist trainees experience a high level of stress at their work, due to the nature of their profession, weaknesses in the health care system, personal traits, inexperience, and interpersonal relations with their colleagues and superiors. An additional burden for them is the needs of their family and/or small children, housework and the obligations of their specialist training. A significant conflict arises from the multitude of these demands resulting in consequences, among which the most alarming is burnout. In spite of the problems they have to face, some of the study participants listed a number of successful coordination tactics. The crucial factor for achieving an effective balance is the empowerment of women doctors, which is enabled through a positive attitude towards self.

### Burdens

Two of the most important burdens in the workplace mentioned by most of the participants was the fast pace and lack of time for patient consultations, combined with great responsibility for patients. A study conducted among 50 general practitioners in Slovenia, which measured overburdening in outpatient clinics, showed that every physician has only 6.93 min on average per patient consultation [19]. The reason lies in the health care system and stems from the lack of general practitioners in Slovenia, resulting in a too many patients per doctor [1]. Specialist trainees in rural general practices pointed out the hardest burdens, and the same applies to other countries [20].

In addition, the participants also emphasised that they carry out patient consultations too slowly and in too much detail. Inexperience, lack of trust in their own judgment and lack of clinical knowledge are also the reasons for slow patient consultations, therefore the feeling of pressure and stress is even more pronounced at work. However, according to other studies, this is characteristic of women doctors in general: women doctors more often stress time pressure at work than their male colleagues, because they spend more time per patient than foreseen [9, 12, 21].

The status of a specialist trainee includes, in addition to inexperience, a lack of autonomy and a subordinate position in relation to senior colleagues and superiors, which often leads to exploitation. This issue is not only recurrent in the literature, but was also clearly voiced by our interviewees, who expressed related helplessness. In North America, exploiting specialist trainees has taken on endemic proportions [2].

There is evidence that personality traits significantly contribute to the development of the burnout syndrome [22, 23]. In this study, we discovered that the trainees perceive themselves as perfectionists and too self-sacrificing, that they find it difficult to say no, and that they do not delegate work appropriately. In their desire to help and be obliging towards their patients and colleagues, they often surpass their own capabilities and the needs of their family.

At home they are faced with the wishes and needs of their children and with housework, which occupies them for the greater part of the remainder of the day. According to the literature, most of the housework is still performed by women, who are also the lead organisers of their home life [7, 9].

It is worthy of noting that they often run out of time for completing their specialisation training obligations, such as modular course assignments and studying.

### Consequences of burdens

The work-home conflict, along with an intensive workload, large quantities of new clinical knowledge and the already mentioned personal traits, are some of the biggest stressors that women doctors encounter [6–10, 24]. They are followed by symptoms of burnout, both physical and mental. Pronounced symptoms of burnout were mentioned especially by the trainees who work in rural clinics and trainees who have an insufficient social support network. The observations on the symptoms of burnout in our participants are consistent with the results of other studies [2, 10, 13–15, 23].

They experience many feelings of guilt. They feel guilty because of the health care work organisation and towards their colleagues when they take sick leave, therefore they try to avoid taking it. They are aware that their absence presents extra stress for other staff. This phenomenon of feeling guilty is also identified in other studies [6, 9].

Feelings of guilt are highest towards their family and children, since they continually lack the time for them, which is often also mentioned in the literature [7–9]. They struggle with feelings of guilt when prioritising their own needs before the needs of their family and children. Their self-care is reduced to a minimum, due to time constraints. They often neglect even the most basic needs, such as rest, sufficient sleep and food, which is also comparable to the data in the literature [6, 8, 9]. This reflects women’s priority systems that place the child first and themselves last.

### Empowerment

Personal empowerment of specialist trainees has been found to constitute the foundation for trainees to be able to find practical solutions and adaptations in their current life/work situation. It showed to be the important mechanism in successful work-family balance. A positive attitude towards self, optimism, a sense of success, coping with stress and self-care are the main factors for empowering women general practitioners. Specialist trainees who find this balance are satisfied in their work and enjoy it. Working is not only a burden for them, but their success at work is also a source of energy for carrying out other burdens.

The two common negative dispositions of women doctors are perfectionism and guilt [9]. Specialist trainees are increasingly aware of this. Their experience and personal growth allow them to take time for themselves with minor feelings of guilt towards their family and children; they are less demanding towards themselves. They pay more attention to catering to their own needs of sleep, regular and healthy eating, and sports. They find support in spending time with friends; a source of relaxation for them is also their hobbies. In the literature, the importance of hobbies and activities outside of medicine is often mentioned in reducing the chances of burnout [25]. They are aware of the importance of good interpersonal relations with their colleagues and they invest their energy in them.

### Coordination

Successful coordination requires physicians to continually set their priorities and adjust their expectations. The same fact has also been established by the WPHS study (women physical health study) [26].

They try to set boundaries within their family life and at work, to suitably delegate work in both. Since it is impossible to satisfy the needs and wishes of everyone, they try to avoid being excessively obliging. Trainee specialists stressed the importance of sensible life choices and effective organisation, both at work and at home.

According to the interviews, coordination in the family would be virtually impossible without the help of a supportive partner, who provides moral support and help with housework, and their parents. The study by Norwegian doctors of both genders has shown that couples where women are physicians have a more equal share of housework compared to other couples [3].

One of the coordination possibilities is an extension of specialisation training, which was not, with one exception, met with approval from the trainees. The new generation of specialist trainees, especially women trainees in other countries, often choose to work part-time as a way of facilitating the coordination of their burdens. Data for Switzerland show that 1/3 of women GP specialist trainees in 2001 benefitted from part-time work. Norway shares a similar trend, where almost half of all women in the general working population are employed on a part-time basis while their children are small [3, 22, 27]. It is evident from the data supplied by the Medical Chamber of Slovenia that there are currently only two women GP specialist trainees working part-time, out of 278, which amount to 1 %. They would take advantage of the possibility to work part-time if it did not have a financial impact and if the difference was covered with funds from the government. The specialist trainees suggested other changes that would facilitate their coordination of burdens. The primary measure would be to employ more general practitioners. This would result in being able to take sick leave and annual paid leave more easily. They wish to be exempt from on-call duty and afternoon shifts. Due to their inexperience, they also wish to have the permanent possibility of consultation with an experienced colleague.

### Strength and limitations of the study

The qualitative research method does not allow for generalisations applied to the whole of the population of women GP specialist trainees, but provides an in-depth insight into the issues of burdens of young doctors.

Phenomenological research in its nature gives interpretation of subjective meaning of human experience, which is interpreted by the researcher according to the data analysis.

The disadvantage of the study might be the fact that the interviews were conducted by a person who was a specialist trainee herself. She was trained in qualitative methodology prior to the study and the questions were carefully chosen within the group of researchers and then tested. After the first few interviews, a discussion with the expert in qualitative methodology (DP) was performed. Nevertheless, it could be a source of bias.

The advantages of the study are an accurately conducted study that adhered to the operative instructions of the qualitative methodology, and an independent analysis performed by two researchers with multiple difference-coordination techniques in the analysis. The sample size of 10 interviews met the requirement of the saturation of data.

Furthermore, the research method provided an in-depth insight into the possibilities and limitations of coordination, and provided potential solutions. The study results could help to improve the position of women GP specialist trainees and reorganise their specialisation training scheme. The results also serve as a warning to GP specialist trainees to be more attentive to their own needs and to the signs and symptoms of burnout.

## Conclusion

The research confirms the fact that women specialist trainees encounter intense burdens at work and home due to numerous demands and requirements during their specialisation training. In addition, there is also the issue of the work–family conflict. Many consequences arise from burden and strain; however, burnout stands out the most. In contrast, reconciliation of work and family life and needs can be successful. The key element is the empowerment of women doctors. Supervisors and physicians involved in the training of specialists should be aware of the complexity of requirements in the specialisation training programme, as well as the accompanying realistic threat of burnout. Systemic and individual solutions are both necessary and feasible, of which the first and foremost is the reinforcement of general practitioners in primary health care and their understanding of the specialisation training scheme, including possible allowable adjustments.
